# A Spanish case-control study in <5 year-old children reveals the lack of association between MLB and VA astrovirus and diarrhea

**DOI:** 10.1038/s41598-020-58691-3

**Published:** 2020-02-04

**Authors:** Diem-Lan Vu, Aurora Sabrià, Nuria Aregall, Kristina Michl, Jaume Sabrià, Virginia Rodriguez Garrido, Lidia Goterris, Albert Bosch, Rosa Maria Pintó, Susana Guix

**Affiliations:** 1 Enteric Virus Laboratory, Department of Genetics, Microbiology and Statistics, University of Barcelona, Barcelona, Spain; 2 Nutrition and Food Safety Research Institute (INSA·UB), University of Barcelona, Barcelona, Spain; 3 Department of Infectious Diseases, Geneva University Hospitals, Geneva, Switzerland; 4 Primary Health Care Center El Serral, Generalitat de Catalunya, Sant Vicenç dels Horts, Spain; 5 Microbiology Department, Hospital Universitari Vall d’Hebron, Barcelona, Spain

**Keywords:** Infectious-disease diagnostics, Viral epidemiology

## Abstract

Novel human astroviruses (HAstV) were discovered 10 years ago and have been associated with fatal cases of central nervous system infections. Their role in gastroenteritis is controversial, as they have been identified in symptomatic and asymptomatic subjects. The aim of the study was to investigate novel HAstV in a gastroenteritis case-control study including a pediatric population in Spain over a one-year period. We included stool samples from patients with gastroenteritis and negative results for viruses screened by routine diagnostics, and stool samples of control subjects who sought for a routine medical consultation. All samples were screened by real-time RT-PCR assays for novel HAstV. An additional screening for rotavirus, norovirus GI, GII, sapovirus, classic HAstV and adenovirus was also performed for the control group. Overall, 23/363 stool samples from case patients (6.3%) and 8/199 stool samples from control patients (4%) were positive for ≥1 novel HAstV. MLB1 was predominant (64.5% of positives). Seasonality was observed for the case group (p = 0.015), but not the control group (p = 0.95). No difference was observed in the prevalence of novel HAstV between the case and control groups (OR 1.78, 95% CI 0.68–5.45; p = 0.30). Nevertheless, MLB genome copy numbers/ml of fecal suspension was significantly higher in the control group than in the case group (p = 0.008). In our study, we identified a lack of association between novel HAstV and gastroenteritis in the studied population, which could indicate a potential role of reservoir for children, especially given the higher viral load observed in the asymptomatic group for some of them.

## Introduction

Human astroviruses (HAstVs) classically cause acute mild to moderate diarrheal illness in young children and the elderly^1^. In immunocompromised hosts, they can cause chronic infections^2–6^ and disseminated disease with severe complications^7^. Recently, several cases of central nervous system infections have been associated with HAstV, mainly the novel clades HMO-VA and MLB^8–14^, which were identified ten years ago^15^. The emergence of these novel clades, which are genetically very distant from the classic HAstV^16^ and are associated with unexpected clinical presentation in humans and also various animal species^17–19^, raises the question of their origin and the pathophysiology of central nervous system infections. Interestingly, a recent study reported the retrospective identification of an astrovirus strain belonging to the HMO-VA clade in a brain biopsy of a muskox that had died of suppurative encephalitis in 1982^20^. Another study suggested a common ancestor between the MLB clade and astrovirus identified in rats^21^. These data suggest a cross-species transmission^22,23^, which could have occurred long time ago. Sequences of novel HAstVs have been found in human stools of individuals with diarrhea^15,24–26^, but also in asymptomatic healthy controls^27,28^; unfortunately, the two latter reports have contradictory results in their case-control study, especially for MLB1, which so far is the most common. We also recently reported a high rate of co-infection with other enteric viruses during episodes of gastroenteritis where a novel astrovirus was identified^29^, raising the question of their causal role in gastroenteritis.

The main aim of the study was to compare the prevalence of novel HAstV in a pediatric population with and without symptoms of gastroenteritis in Spain, in order to assess the association of novel HAstVs with acute gastroenteritis in children. In addition, we also aimed at characterizing the prevalence of infection by novel HAstVs as well as other common enteric viruses among asymptomatic children and determining the risk factors for being asymptomatically infected.

## Results

### Case patients

**Figure 1:**
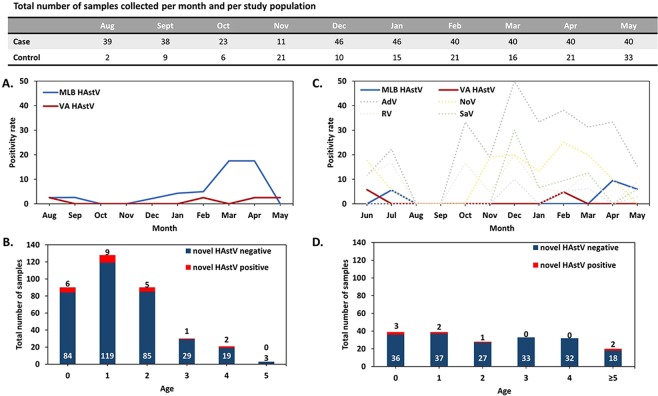
Prevalence of novel astrovirus and other viruses according to month of sampling and age groups. Prevalence in the case population according to month of sampling (**A**) and age groups. (**B**) The table indicates the absolute number of samples collected per month and per study population. Prevalence in the control population according to month of sampling (**C**) and age groups. (**D**) Numbers above the bar correspond to the absolute number of positive samples for novel HAstVs, numbers at the bottom of the bar correspond to the absolute number of negative samples for novel HAstVs. Two patients in the control population had 6 years old, the screening for novel HAstVs was positive in one and negative in the other. HAstV: Human astrovirus; AdV: Adenovirus; NoV: Norovirus; RV: Rotavirus; SaV: Sapovirus.

Among the 363 stool samples analyzed from children with acute gastroenteritis living in the Barcelona Metropolitan Area, collected between August 2017 and May 2018, 23 were positive for a novel HAstV (6.3%), including 21 MLB (16 MLB1, 5 MLB2-3) and 4 VA (1 VA1, 1 VA2, 2 VA3). Two patients had a double infection (MLB2 + VA1 and MLB1 + VA3). Median log_10_ RNA copy numbers/ml of fecal suspension were 2.35 (IQR 2.13–3.76) and 3.23 (IQR 2.87–8.86) for HAstV-MLB and HAstV-VA, respectively Thirty-five out of 363 (9.6%) patients had a positive coproculture. There was no significant difference in the prevalence of novel HAstVs between patients with (3/35, 8.6%) and without (20/328, 6.1%) a positive coproculture (p = 0.48), but the median log_10_ RNA copy numbers/ml of fecal suspension was statistically higher in patients with a positive coproculture (5.19, IQR 4.24–6.22) than in those with a negative coproculture (2.31, IQR 2.11–3.32) (p = 0.02). The prevalence of novel HAstV differed significantly between month (p = 0.015) (Fig. 1A), while there was no difference according to age (p = 0.92), or age group categorized as ≤1 year old versus >1 year old (p = 0.66) (Fig. 1B).

### Control patients

A total of 199 children were included between June 2017 and May 2018. Eight were positive for a novel HAstV (4%), including 5 MLB (4 MLB1, 1 MLB2-3) and 3 VA (1 VA1, 1 VA2, 1 VA3). There were no co-infections observed with several novel HAstV. Median log_10_ RNA copy numbers/ml of fecal suspension were 6.52 (IQR 4.52–6.84) and 2.70 (IQR 1.73–9.05) for HAstV-MLB and HAstV-VA, respectively. The prevalence of novel HAstV did not significantly differ between month (p = 0.95) (Fig. 1C), nor between age (p = 0.06) (Fig. 1D), sex (p = 0.75), the presence of a chronic pathology (p = 1.0), breastfeeding (p = 1.0), rotavirus complete vaccination (p = 0.14), use of antibiotics (p = 0.14) or probiotics (p = 1.0) use in the preceding month, the presence of fever or respiratory symptoms (p = 0.72) in the preceding 2 weeks, the presence of siblings (p = 0.27), school or daycare center attendance (p = 1.0), exposition to domestic pets (p = 1.0), or the presence of any other enteric virus (p = 0.73) (Table 1).

**Table 1:** Risk factors for asymptomatic infection by novel HAstVs and other enteric viruses in children (2017–2018).

Risk Factor	Virus Prevalence (%)	OR	95% CI	P value
**Novel HAstV**				
Age (0–1 year/2–6 years)	6.4/2.7	2.5	0.47–16.6	0.28
Gender (Boy/Girl)	4.7/3.4	1.37	0.26–9.08	0.73
Breastfeeding (Yes/No)	4.7/8.7	0.53	0.008–10.98	1.00
Chronic Pathology (Yes/No)	0.0/4.6	—	—	1.00
RV Vaccination (Yes/No)	6.3/1.9	3.55	0.56–37.94	0.14
Antibiotic Treatment (Yes/No)	12.5/3.4	4.02	0.36–25.12	0.14
Resp infection/Fever (Yes/No)	5.1/3.5	1.5	0.27–8.30	0.72
Siblings (Any/None)	4.5/3.4	1.33	0.23–13.90	1.00
School Attendance (Yes/No)	4.4/3.7	1.20	0.23–7.97	1.00
Domestic Animals (Yes/No)	3.9/4.3	0.92	0.16–5.08	1.00
**Adenovirus**				
Age (0–1 year/2–6 years)	19.2/30.4	0.54	0.25–1.14	0.09
Gender (Boy/Girl)	25.5/27.6	0.89	0.45–1.8	0.74
Breastfeeding (Yes/No)	0/34.8	—	—	**0**.**004**
Chronic Pathology (Yes/No)	25/27.2	0.89	0.15–3.79	1.00
RV Vaccination (Yes/No)	24.1/28.3	0.80	0.39–1.64	0.61
Antibiotic Treatment (Yes/No)	18.8/27	0.62	0.11–2.42	0.56
Resp infection/Fever (Yes/No)	24.7/27.8	0.85	0.41–1.72	0.74
Siblings (Any/None)	25.6/27.1	0.92	0.44–1.98	0.86
School Attendance (Yes/No)	30.4/19.8	1.77	0.86–3.75	0.13
Domestic Animals (Yes/No)	26.7/25	1.09	0.55–2.2	0.87
**Norovirus**				
Age (0–1 year/2–6 years)	13.2/10	1.36	0.49–3.76	0.64
Gender (Boy/Girl)	10.5/13.1	0.77	0.29–2.10	0.65
Breastfeeding (Yes/No)	9.5/19.1	0.45	0.04–3.65	0.66
Chronic Pathology (Yes/No)	25/9.5	3.16	0.49–14.37	0.12
RV Vaccination (Yes/No)	20.8/4.8	5.19	1.69–18.89	**0**.**002**
Antibiotic Treatment (Yes/No)	25/10	3	0.63–11.34	0.08
Resp infection/Fever (Yes/No)	14.5/9.8	1.55	0.57–4.19	0.36
Siblings (Any/None)	9.9/15.8	0.59	0.22–1.67	0.32
School Attendance (Yes/No)	11.7/11.4	1.03	0.38–2.89	1.00
Domestic Animals (Yes/No)	10.2/13.2	0.74	0.27–2.00	0.65
**Rotavirus**				
Age (0–1 year/2–6 years)	19.2/1.8	13.2	2.89–121.3	** < 0**.**001**
Gender (Boy/Girl)	7.5/10.3	0.7	0.22–2.15	0.61
Breastfeeding (Yes/No)	38.1/17.4	2.92	0.61–15.8	0.18
Chronic Pathology (Yes/No)	8.3/9.2	0.89	0.02–6.9	1.00
RV Vaccination (Yes/No)	10.1/7.5	1.39	0.43–4.48	0.6
Antibiotic Treatment (Yes/No)	0/9.7	—	—	0.37
Resp infection/Fever (Yes/No)	3.8/12.2	0.29	0.05–1.09	0.07
Siblings (Any/None)	6.7/10.2	0.64	0.19–2.29	0.39
School Attendance (Yes/No)	2.7/17.3	0.13	0.02–0.5	**0**.**001**
Domestic Animals (Yes/No)	5.9/11.8	0.47	0.14–1.47	0.2
**Sapovirus**				
Age (0–1 year/2–6 years)	7.7/2.7	3.05	0.63–19.36	0.16
Gender (Boy/Girl)	5.6/3.4	1.66	0.34–10.6	0.7
Breastfeeding (Yes/No)	4.7/8.7	0.53	0.008–11.0	1.00
Chronic Pathology (Yes/No)	8.3/3.5	2.53	0.05–23.84	0.38
RV Vaccination (Yes/No)	6.3/3.7	1.74	0.36–9.05	0.49
Antibiotic Treatment (Yes/No)	0/5.1	—	—	1.00
Resp infection/Fever (Yes/No)	7.7/1.7	4.7	0.81–48.54	0.06
Siblings (Any/None)	4.5/5.1	0.88	0.18–5.6	1.00
School Attendance (Yes/No)	6.2/2.5	2.61	0.48–26.3	0.3
Domestic Animals (Yes/No)	3.9/5.4	0.73	0.14–3.49	0.7
**Classic HAstV**				
Age (0–1 year/2–6 years)	6.4/0.9	7.6	0.82–362	0.04
Gender (Boy/Girl)	4.7/1.2	4.2	0.45–199	0.2
Breastfeeding (Yes/No)	0/13.0	—	—	0.23
Chronic Pathology (Yes/No)	0/2.9	—	—	1.00
RV Vaccination (Yes/No)	6.4/0.9	7.3	0.78–346	0.08
Antibiotic Treatment (Yes/No)	6.3/2.3	2.83	0.05–30.9	0.4
Resp infection/Fever (Yes/No)	3.8/2.6	1.48	0.19–11.32	0.7
Siblings (Any/None)	3.0/1.7	1.8	0.17–90.1	1.00
School Attendance (Yes/No)	4.4/1.3	3.53	0.38–169	0.4
Domestic Animals (Yes/No)	3/3.2	0.93	0.12–7.12	1.00

Only complete vaccinated individuals were included in Yes for rotavirus vaccination.

Breastfeeding Yes includes partial and total breastfeeding of children under 1 year-old. OR: odds ratio; CI: confidence interval; RV: rotavirus.

Control children were also screened for other common enteric viruses using qualitative commercial assays and viral titers were assessed by using the Cq values. We found 52 adenoviruses (26.1%; median Cq 35.5, IQR 32.5–39.2), 22 noroviruses (11%; median Cq 35, IQR 30.5–38.1), including 5 GI and 17 GII noroviruses, 17 rotaviruses (8.5%; median Cq 32.7, IQR 30.1–37.3), 10 sapoviruses (5%; median Cq 30.7, IQR 26.3–34.1), and 7 classic HAstVs (3.5%; median Cq 37.2, IQR 31.6–38.2) (Fig. 1C). Co-infections were encountered in 5 out of 8 novel HAstV-positive patients with the following viruses: norovirus GI (n = 1), classic HAstV (n = 1), rotavirus (n = 1), adenovirus (n = 3). One of these patients had co-infection with 3 viruses (VA3, norovirus GI and adenovirus). There was no statistically significant difference of Cq values between virus groups (p = 0.07). The prevalence of other enteric viruses did not significantly differ between month (adenovirus p = 0.14; norovirus p = 0.15; rotavirus p = 0.59; sapovirus p = 0.10; classic HAstV p = 0.68). According to other potential risk factors analyzed, we found that breastfeeding was protective for adenovirus infection (p = 0.004) and children with rotavirus vaccination had a higher odd of norovirus infection (OR 5.19, CI 1.69–18.89; p = 0.002). Children older than one year old and school attendance were found to be protective for rotavirus infection, but after logistic regression, only age remained statistically significant (p = 0.02) (Table 1).

### Comparison between case and control groups

Globally there was no statistically significant difference in the prevalence of novel HAstV between the case and the control population (OR 1.78, 95% CI 0.68–5.45; p = 0.30) (Table 2). By analyzing MLB and VA clade separately, we also found no differences between cases and controls (MLB: OR 2.46, 95% CI 0.80–9.98; p = 0.12. VA: OR 0.90, 95% CI 0.13–10.07; p = 1.00), and the same was true when only MLB1, the most frequently detected, was considered (OR 2.47, 95% CI 0.69–13.42; p = 0.20). Nevertheless, while the log_10_ RNA copy numbers/ml of fecal suspension did not significantly differ between case and control subjects (p = 0.07), it was the case for MLB when analyzing both clades separately (p = 0.008) (Table 1). Unexpectedly, log_10_ RNA copy numbers/ml of fecal suspension were higher in the control subjects than the case subjects. Using logistic and linear regression to adjust novel HAstV positivity rate and MLB log_10_ RNA copy numbers/ml of fecal suspension obtained in case versus control population for confounding factors such as astrovirus clade, the month of sample collection and patient’s age (Table 2), the difference in the positivity rate remained non-significant (p = 0.14), while the MLB log_10_ RNA copy numbers difference remained statistically significant (p = 0.008).

**Table 2:** Characteristics of the case and control populations.

	Case (n = 363)	Control (n = 199)	P Value	OR (95% CI)
Sex (male), n (%)	210 (58)	107 (55.2)	0.5	
• Missing value	1	5		
Age, mean (SD)	1.37 (1.16)	2.21 (1.68)	**<0.001**	
• Missing value	1	8		
Number of samples collected according to month, n (%)			**<0.001**	
• Jan-Feb	86 (23.7)	36 (18.1)		
• Mar-Apr	80 (22.0)	37 (18.6)		
• May-Jun	40 (11.0)	50 (25.1)		
• Jul-Aug	39 (10.7)	20 (10.1)		
• Sept-Oct	61 (16.8)	15 (7.5)		
• Nov-Dec	57 (15.7)	31 (15.6)		
• Missing value	0 (0)	10 (5.0)		
Positive novel HAstV, n (%)*	23 (6.3)	6 (3.7)	0.30	1.78 (0.68–5.45)
• Log_10_ RNA copies/ml, median (IQR)	2.54 (2.18–3.76)	6.22 (2.81–7.66)	0.07	
Positive for MLB, n (%)*	21 (5.8)	4 (2.4)	0.12	2.45 (0.81–9.98)
• Log_10_ RNA copies/ml, median (IQR)	2.35 (2.13–3.76)	6.52 (4.52–6.84)	**0.008**	
Positive for VA, n (%)*	4 (1.1)	2 (1.2)	1.00	0.90 (0.13–10.07)
• Log_10_ RNA copies/ml, median (IQR)	3.23 (2.87–8.86)	2.70 (1.73–9.05)	0.47	

P value for comparison between case and control patients. *The prevalence calculated here included the period between August-May in order to appropriately compare case and control patients. RNA copies correspond to RNA copy number/ml of fecal suspension. OR: odds ratio; CI: confidence interval; IQR: interquartile range; Jan: January; Feb: February; Mar: March; Apr: April; Jun: June; Aug: August; Sept: September; Oct: October; Nov: November; Dec: December; HAstV: Human astrovirus.

**Figure 2:**
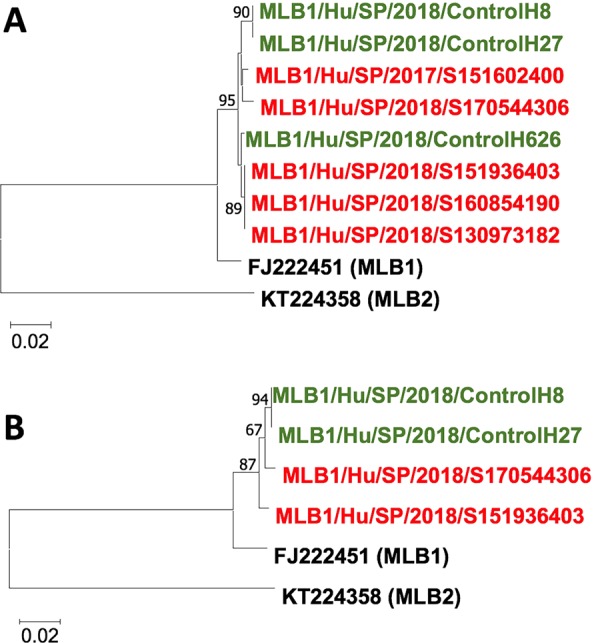
Phylogenetic trees of MLB1 human astroviruses based on amplicon sequences of the capsid coding gene (357 bp) (**A**) and the RNA polymerase gene (370 bp) (**B**). Trees were constructed by Neighbor-Joining method with 500 bootstrap replicates. The evolutionary distances were computed using the p-distance method and are in the units of the number of base differences per site. Reference sequences for MLB1 and MLB2 HAstVs are denoted with a black color; sequences from control children are indicated in green, and sequences from children with gastroenteritis are denoted in red. Sequences obtained in the study were deposited in GenBank (accession numbers MN689595-MN689606).

Sequence information was obtained from selected MLB1 strains infecting control subjects as well as children with gastroenteritis, using previously described primers^15^, in order to analyze whether circulating strains were specific to each group. Analysis of a partial capsid-coding sequence (357 bp) revealed that although sequences isolated from controls and cases were not identical, strains infecting controls and cases were genetically closely related (Fig. 2). Analysis of a partial sequence based on the RNA polymerase RNA dependent coding gene (370 bp) did not suggest the occurrence of recombinant strains. Sequences were deposited in Genbank (accession numbers MN689595-MN689606).

## Discussion

In the present study, we demonstrated that novel HAstV-MLB and HAstV-VA may be identified at similar frequencies in symptomatic and asymptomatic children up to 5 years old. This important finding argues in favor of the potential for young children to act as reservoir for transmission of novel HAstV, which is of most public health concern, regarding central nervous system complications that can occur, mostly in immunocompromised patients^16^.

We found that 6.3% of case patients were positive for one or several novel HAstV. This is slightly lower than our previous study, which investigated a similar Spanish pediatric population, with a positivity rate of 10%^29^. The difference can be explained by the study period: our first study focused on two consecutive winter seasons from January-April 2016 and January-April 2017, respectively, while the present one extended over a 10-months period (August 2017-May 2018). Yet, a statistically significant difference in novel HAstV prevalence according to the month of sample collection could be observed here, with January-April months being the ones with the highest detection rate. This seasonality is in agreement with the study of Jacobsen *et al*.^30^, although the latter focused on both novel and classic HAstV.

The novel HAstV positivity rate among control subjects was 4%. This is comparable to findings in the Indian cohort of Holtz *et al*.^28^ and the Gambian cohort of Meyer *et al*.^27^. We could not identify factors predicting the asymptomatic carriage of novel HAstV in children, but we demonstrated that asymptomatic children can harbor a high variety of eukaryotic viruses, with adenovirus and norovirus being the most frequently identified. In a case-control study screening the presence of several enteric pathogens by conventional PCR assays, the global positivity rates for norovirus, rotavirus A and sapovirus in control subjects were 16%, 14%, and 2%, respectively^31^, which is in accordance with our results. Rotavirus is classically associated with severe gastroenteritis in infants^32^, but asymptomatic infections can occur^33^, and may reflect an infection during the period of maternal antibodies protection or a reinfection with a previously mounted immune response allowing to control/prevent the disease^33^. Additionally, there could be a lower susceptibility to rotavirus infection correlated with the genetic variability of the histo-blood group antigen (HBGA) cell receptor^33^. Surprisingly, we found no association between rotavirus asymptomatic infection and vaccination status, but this could be due to the small number of rotavirus infections. After logistic regression, only children between 0 and 1 year old were significantly at increased risk for rotavirus infection, which is consistent with the lack of immunity in this population group^32^. As for norovirus, asymptomatic shedding in children has been reported to be in the range from <1% to >30% in different European and non-European countries^34–38^. The higher prevalence of norovirus in patients vaccinated for rotavirus could be explained by a shift in the type of viral infection as has been observed in patients in certain countries where rotavirus vaccination has been implemented^39–43^.

No difference in the prevalence of novel HAstV between the case and the control groups was observed. While Holtz *et al*. did not find any association between MLB1 infection and diarrhea^28^, Meyer *et al*. reported an association between MLB1 and diarrhea and between MLB3 and asymptomatic disease, and no significant association of VA viruses with any of the groups^27^. The results of the present study are also in agreement with the high rate of co-infections observed in our previous study: MLB and VA were identified in co-infection with ≥1 other enteric virus in 78% and 54.8% of cases (all symptomatic), respectively, raising the question of their contribution to the digestive tract symptoms^29^. In this previous study, we could not observe a difference in the novel HAstV mean viral RNA titer during mono- and co-infection. In the present study, the RNA concentration in fecal suspensions significantly differed between both groups, particularly for the MLB clade, and was unexpectedly higher in asymptomatic infections than during episodes of gastroenteritis. This interesting finding points at a potential mechanism of protection towards other pathogenic enteric viral infections: the presence of MLB HAstV at higher viral load in asymptomatic patients could confer a protection due to viral interference^39,40^. Our hypothesis is supported by a recent study by Ingle *et al*., who demonstrated that a specific strain of murine HAstV was able to protect immunodeficient mice from infection by murine norovirus and rotavirus through high level of IFNλ 2/3, without inducing intestinal inflammation^41^. In addition, novel astroviruses were identified at higher viral load in presence of a pathogenic bacteria, raising the question of whether astrovirus could influence the equilibrium of the gut microbiome^42^.

Our study has some limitations: we could not perform a full one-year screening of the case patients, which could have influenced our analysis of seasonality. In the control group, sample collection varied from one month to another probably due to the criteria of exclusion (gastroenteritis, age), the randomness of the pediatric healthy controls and holiday periods. Yet, few samples could be collected during months of August through October. Our study also focused on a one-year period and we cannot exclude that there is a variability in astrovirus prevalence from one year to another, as previously suggested^43^. Therefore, our analysis of seasonal pattern of novel astrovirus has to be confirmed by larger studies extending over several years, and our conclusions are so far limited to the 2017/2018 period. Also, according to the mentioned difficulty to recruit healthy controls, it is still possible that our study was underpowered to demonstrate a difference of prevalence between both groups, but the higher viral load in the asymptomatic group supports the lack of association with diarrhea. Finally, a deep genomic analysis to compare strains infecting asymptomatic and diarrheic children will be performed in future studies to assess whether molecular determinants of symptomatic or asymptomatic infections may exist.

In conclusion, our study provides additional information on novel HAstV epidemiology among asymptomatic children and reinforces the hypothesis that these emerging viruses are not necessarily associated with gastrointestinal syndromes. The unexpected finding that viral load is higher among asymptomatic than symptomatic children and in presence of pathogenic bacteria should raise our attention on the potential role of novel HAstV in the intestinal homeostasis and drive further investigations to understand the mechanisms underlying severe complications.

## Methods

### Patient and sample population

The case study group consisted of children ≤5 years old attending outpatient clinics in the Barcelona Metropolitan Area for symptoms of gastroenteritis. Stool samples were analyzed for routine diagnostics at the Microbiology Laboratory of Hospital de Vall d’Hebron, Barcelona (Spain). No clinical data were available except the patients’ age and the date of stool collection. Stool samples positive for parasites, classic HAstV, rotavirus or adenovirus by routine diagnostics, including immunochromatographic tests for viruses, were excluded. We decided not to exclude bacteria, according to evidence for transkingdom interactions between some enteric viruses, including astroviruses, and intestinal bacteria^42,44^. A total of 363 stool samples were randomly selected between August 2017 and May 2018. The study was conducted in accordance with the Declaration of Helsinki, and was approved by the Clinical Research Ethic Committee of the Hospital Universitari Vall d’Hebron (PR(AG)32/2016; February 2016).

The control study group consisted in children of ≤5 years old visiting the primary health care center El Serral, Sant Vicenç dels Horts, Barcelona (Spain) for their routine control visits. Patients were excluded in the study if they had shown symptoms of gastroenteritis such as diarrhea or vomiting during the preceding 4 weeks. The following clinical and demographic data were collected: age, sex, date of sample collection, any chronic pathology, breastfeeding history, rotavirus vaccination status, antibiotic or probiotic use in the preceding month, fever and/or respiratory symptoms in the preceding 2 weeks, siblings, school or daycare center attendance, and domestic pet exposition. Parents or legal representatives of all patients signed an informed consent before being included in the present study and the study was approved by the Clinical Research Ethic Committee of the Institut Universitari d’Investigació en Atenció Primària Jordi Gol (P17/095; May 2017).

### Viral RNA extraction and real-time reverse transcription-polymerase chain reaction (real-time RT-PCR) assays for novel HAstV and other enteric viruses

All samples were screened for the presence of novel HAstVs. RNA was extracted from 200 µl of a 30% stool suspension in phosphate buffered saline (PBS) using the Viasure RNA-DNA extraction kit (Certest Biotec) following the manufacturer’s instructions. Real-time RT-PCR specific assays for HAstV-MLB and HAstV-VA were performed in duplex as previously described^29,45^, using the Kapa Probe Fast Universal One-Step real-time RT-PCR Master Mix (Kapa Biosystems) and following the manufacturer’s instructions, on a Stratagene Mx3000P (Thermofischer) and a CFX96 Touch™ Real-Time PCR Detection System (Bio-Rad). The standard curves for quantification were constructed based on 10-fold serial dilutions of plasmids containing a fragment of the corresponding genome strains (MLB1: nucleotides 4292–4416 from FJ222451; MLB2: nucleotides 3724–3848 from KT224358; VA1: nucleotides 4629–4706 from FJ973620.1; VA2: nucleotides 4480–4558 from GQ502193.2; VA3: nucleotides 3678–3756 from JX857868.1; VA4: nucleotides 4646–4721 from JX857869.1).

The presence of other enteric viruses in stool samples of the control population were also assayed by real-time RT-PCR assays for classical HAstVs, sapovirus, rotavirus, adenovirus, and noroviruses GI and GII, using commercial kits (VIASURE Astrovirus Real Time PCR Detection Kit, VIASURE Sapovirus Real Time PCR Detection Kit, VIASURE Adenovirus Real Time PCR Detection Kit, VIASURE Norovirus GI Real Time PCR Detection Kit, VIASURE Norovirus GII Real Time PCR Detection Kit, and VIASURE Rotavirus Real Time PCR Detection Kit, from Certest Biotec). This additional screening was performed in order to complete the information on the asymptomatic carriage of novel astrovirus in healthy children, providing a comparison with prevalence of mostly encountered enteric viruses. We did not performed such complementary screening among the diarrheic group, as this was already done in a previous study^29^.

### Nucleotide sequencing and phylogenetic analysis

Sequence information was obtained after amplification by RT-PCR using SF0073-SF0076 and SF0053-SF0061 primers, targeting the conserved RNA polymerase region and the MLB1 capsid coding region, respectively^15^. Nucleotide sequences were used for the construction of a phylogenetic tree by the neighbor-joining method using MEGA7.0^46^.

### Statistical analyses

The Mann-Whitney and Kruskal Wallis tests were used to compare continuous variables and Fischer exact test or Chi2 test were used to compare categorical variables. Linear and logistic regression analyses were performed to adjust for confounding variables. P < 0.05 was considered statistically significant. Statistics were performed by Stata/IC 13.1 (StataCorp, College Station, TX, USA).

## Data Availability

The datasets generated during and/or analysed during the current study are available from the corresponding author on reasonable request.
